# Evaluation of Disinfectant Efficacy against Biofilm-Residing Wild-Type *Salmonella* from the Porcine Industry

**DOI:** 10.3390/antibiotics12071189

**Published:** 2023-07-14

**Authors:** Ane Mohr Osland, Claire Oastler, Katharina Konrat, Live L. Nesse, Emma Brook, Anja M. Richter, Rebecca J. Gosling, Mardjan Arvand, Lene K. Vestby

**Affiliations:** 1 Department of Analysis and Diagnostics, Norwegian Veterinary Institute (NVI),1433 Ås, Norway; 2 Department of Bacteriology, Animal and Plant Health Agency (APHA), Weybridge KT15 3NB, UK; claire.oastler@apha.gov.uk (C.O.); emma.brook@apha.gov.uk (E.B.); becky.gosling@hse.gov.uk (R.J.G.); 3 Hospital Hygiene, Infection Prevention and Control, Department Infectious Diseases Robert Koch Institute (RKI), 13353 Berlin, Germany; konratk@rki.de (K.K.); richteranj@rki.de (A.M.R.); arvandm@rki.de (M.A.); 4 Department of Food Safety and Animal Health, Norwegian Veterinary Institute (NVI), 1433 Ås, Norway; live.nesse@vetinst.no; 5 Health and Safety Executive, The Science and Research Centre, Derbyshire SK17 9JN, UK; 6 Department of Infectious Diseases, Medical Microbiology and Hygiene, University of Heidelberg, 69120 Heidelberg, Germany

**Keywords:** biofilm, *Salmonella*, disinfection, pig industry, glutaraldehyde, peracetic acid, biocide, zoonosis, food safety

## Abstract

*Salmonella enterica* is a causative pathogen of Salmonellosis, a zoonosis causing global disease and financial losses every year. Pigs may be carriers of *Salmonella* and contribute to the spread to humans and food products. *Salmonella* may persist as biofilms. Biofilms are bacterial aggregates embedded in a self-produced matrix and are known to withstand disinfectants. We studied the effect of glutaraldehyde and peracetic acid, two active substances frequently used in disinfectant formulations in the pig industry, on representative biofilm-residing wild-type *Salmonella* collected from pig housings in the United Kingdom (UK). We screened biofilm production of strains using the microtiter plate (MTP) assay and Congo Red Coomassie Blue (CRCB) agar method. Previously published stainless-steel coupon (SSCA), polyvinylchloride coupon (PCA), and glass bead (GBA) assays were used for disinfection studies. The mean reduction in the tested wild-type strains met the criterion of ≥4 log_10_ CFU at a disinfectant concentration of 0.05% with SSCA and GBA, and 0.005% with PCA for peracetic acid, along with 0.5% for glutaraldehyde with all three assays on the mean. At these concentrations, both tested disinfectants are suitable for disinfection of pig housings against *Salmonella*. When evaluating the efficacy of disinfectants, biofilms should be included, as higher disinfectant concentrations are necessary compared to planktonic bacteria.

## 1. Introduction

Salmonellosis was the second most reported zoonotic disease in the European Union (EU) in 2020. The overall economic burden of human salmonellosis may be close to EUR 3 billion annually, and in 2021, there were 60,050 reported cases in the EU [1]. Salmonellosis is caused by *Salmonella enterica*, a bacterium that affects animals as well as humans. In humans, it can cause bacteremia and sepsis as well as milder symptoms, such as abdominal cramps, fever, and diarrhea. Transmission occurs when bacteria are shed from the intestines of asymptomatic or sick animals and can infect humans through the consumption of animal products, such as raw meats and eggs. Pigs are considered a possible reservoir and may be asymptomatic carriers for *Salmonella enterica* subspecies *enterica* serovars, such as *S. typhimurium* and *S. derby* [2,3,4]. *S*. Typhimurium was the second most common *Salmonella* serovar involved in human infections in 2021 [1] and the serovar most often responsible for food poisoning [5]. It may also be multi-drug-resistant [6,7].

Spread of infection may occur by direct or indirect contact through the environment. *Salmonella* may survive in production facilities, and although its optimal temperature is 37 °C, the bacteria are able to proliferate between 8 and 45 °C, at pH range 4–9.5, and under conditions of low water activity [8]. This suggests they can adapt to conditions in the food-producing chain, which is also consistent with previous studies [9,10,11]. *Salmonella* is adept to persisting in the environment due to its ability to form biofilms, a correlation found by several scientific authors [12,13,14,15]. In biofilms, bacteria form aggregates and are protected in a self-produced matrix composed of proteins, polysaccharides, and extracellular DNA [16,17,18].

Biofilms are the preferred form of life for bacteria in the environment. Certain surroundings in pig housings are especially prone to the development of biofilms (water pipes, feeding troughs, water troughs, and cracks and crevices) as these areas are more difficult to clean or infrequently cleaned [19]. Bacteria in biofilms are more tolerant to disinfectants than when in their free-living state. The required ≥4 log_10_ colony forming units (CFUs) reduction in bacteria stated in the European disinfection standards (EN 13697:2015+A1:2019 [20]) is more difficult to reach when bacteria are associated with a biofilm [20,21,22,23], and it has also been shown that higher concentrations of disinfectant are required to achieve a ≥5 log_10_ CFU reduction in *Salmonella* reference strains in biofilms [24].

*Salmonella* biofilms can be classified according to their colony morphology on Congo Red Coomassie Blue (CRCB) agar plates and the character of certain extracellular elements [12,17,25] in the matrix. These matrix elements are thin aggregative fimbriae (curli) and cellulose. The red, dry, and rough (RDAR) morphology is found in strains that produce both. Those with brown, dry, and rough (BDAR) morphology produce only curli fimbriae, whereas those with pink, dry, and rough (PDAR) morphology produce only cellulose. Strains that develop into colonies with smooth and white (SAW) morphology produce neither curli fimbriae nor cellulose [17,25]. The level of biofilm production can be measured in the crystal violet microtiter plate (MTP) assay, which is a fast and efficient method of screening multiple strains in a short amount of time [12].

In the pig industry, disinfectants are used as part of the cleaning and disinfection regime in pig housing. Cleaning takes place before disinfection to mechanically remove organic and inorganic matter that may interfere with disinfectant activity [13]. Previous studies have shown that there are differences between classes of disinfectants in their ability to combat *Salmonella* isolated from pig environments [26]. In general, disinfectants have a wide spectrum of activity and may potentially have several microbial targets, making them advantageous in the cleaning of animal housings. However, their efficacy may be reduced upon contact with components, such as organic matter and biofilm-associated pathogens [27,28].

As such, it is important to consider several factors when designing a disinfection strategy for animal housings. The disinfectant class together with concentration and exposure time, the bacterial species to be targeted, and the age and the morphology of biofilm are all aspects that should be considered. There are several disinfectant efficacy assays published for studying environmental biofilms; however, there is not an agreed standard. The assays all accommodate different conditions (e.g., surface, age of biofilm, shear forces, biofilm formed below or at air–surface interface). Thus, it is important to assess the practical application of the test to ensure its relevance.

The aim was to study the effect of disinfectants on representative wild-type *Salmonella* strains from the pig industry in biofilms using three different previously published assays: stainless-steel coupon assay (SSCA), polyvinylchloride coupon assay (PCA), and glass bead assay (GBA) [25]. Two active ingredients frequently included in disinfectant formulas, glutaraldehyde and peracetic acid, were tested. Test strains were selected based on the screening of biofilm production and colony morphology of strains from the pig industry. The study was part of the One Health European Joint Project (OHEJP) BIOPIGEE.

## 2. Results

### 2.1. Screening of Strains from the Pig Industry for Biofilm Production, Morphotypes, and Phage Typing

The wild-type strains (*n* = 50) displayed a large variation in biofilm production in microtiter plates, with OD_600_ varying from 0.037 to 1.213 (Tables S1 and S2). The mean biofilm production of *S*. Typhimurium strains was significantly higher than that of *S*. Derby strains (Table 1). No significant difference was observed between monophasic *S*. Typhimurium and other *S*. Typhimurium strains (0.848 ± 0.272 vs 0.827 ± 0.334, respectively, *p* = 0.85). The biofilm production in the microtiter plate assay (MTP) by strains with RDAR morphology was significantly higher than that of strains with BDAR or SAW morphology (Table 2). Phage type did not influence biofilm production in the MTP assay under the conditions tested. For information on separate strains, see Tables S1 and S2 in the Supplementary Materials.

### 2.2. Selection of Strains

A total of six strains were selected for the disinfection experiments (Table 3). Five strains, four *S*. Typhimurium (including monophasic variants) and one *S.* Derby, were chosen because they expressed RDAR morphology and displayed high OD_600_ in the microtiter plate assay. In addition, one *S*. Typhimurium strain that expressed the BDAR morphotype and a low OD_600_ was included.

### 2.3. Correlation between Biofilm Formation Capacity in the MTP Assay and Achievable Cell Counts in Coupon/Bead Assays

The mean biofilm production of the six selected wild-type test strains without disinfection in the three coupon/bead assays was 6.76 ± 0.28, 6.32 ± 0.45, and 7.58 ± 0.54 log_10_ CFU per biofilm/mL ± SD in the SSCA, the PCA, and the GBA, respectively. The means were significantly different (Student’s *t*-test *p* < 0.05) to each other.

**Figure 1:**
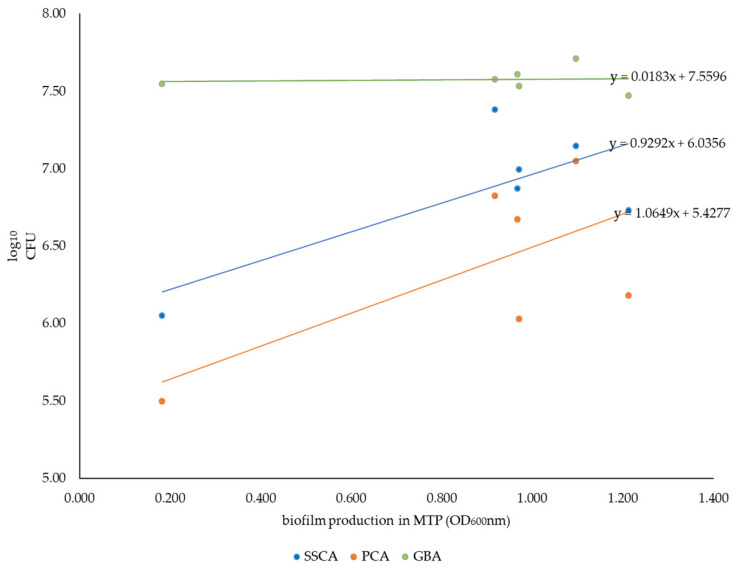
Correlation (indicated by trend line and mathematical formulas) between biofilm production in the MTP assay and the three assays (SSCA, PCA, GBA) for all strains selected for the disinfection experiments.

A positive correlation in the SSCA and the PCA between the amount of biofilm in the coupon assays (given in CFU/biofilm) and the amount of biofilm produced in the MTP assay (specified in OD_600_ of bound Crystal Violet (CV) for each strain) was indicated by the correlation formula, as seen in the Figure 1. In contrast, the same correlation was not present with the GBA, where the amount of biofilm (here as achievable CFU/mL) was consistently high for all strains tested and did not vary depending on the colony morphology or amount of biofilm produced in the MTP assay.

### 2.4. Effect of Disinfection on Biofilm of Selected Wild-Type Strains

#### 2.4.1. Achievable CFU per Biofilm/mL of Separate Strains Following Disinfection

**Figure 2:**
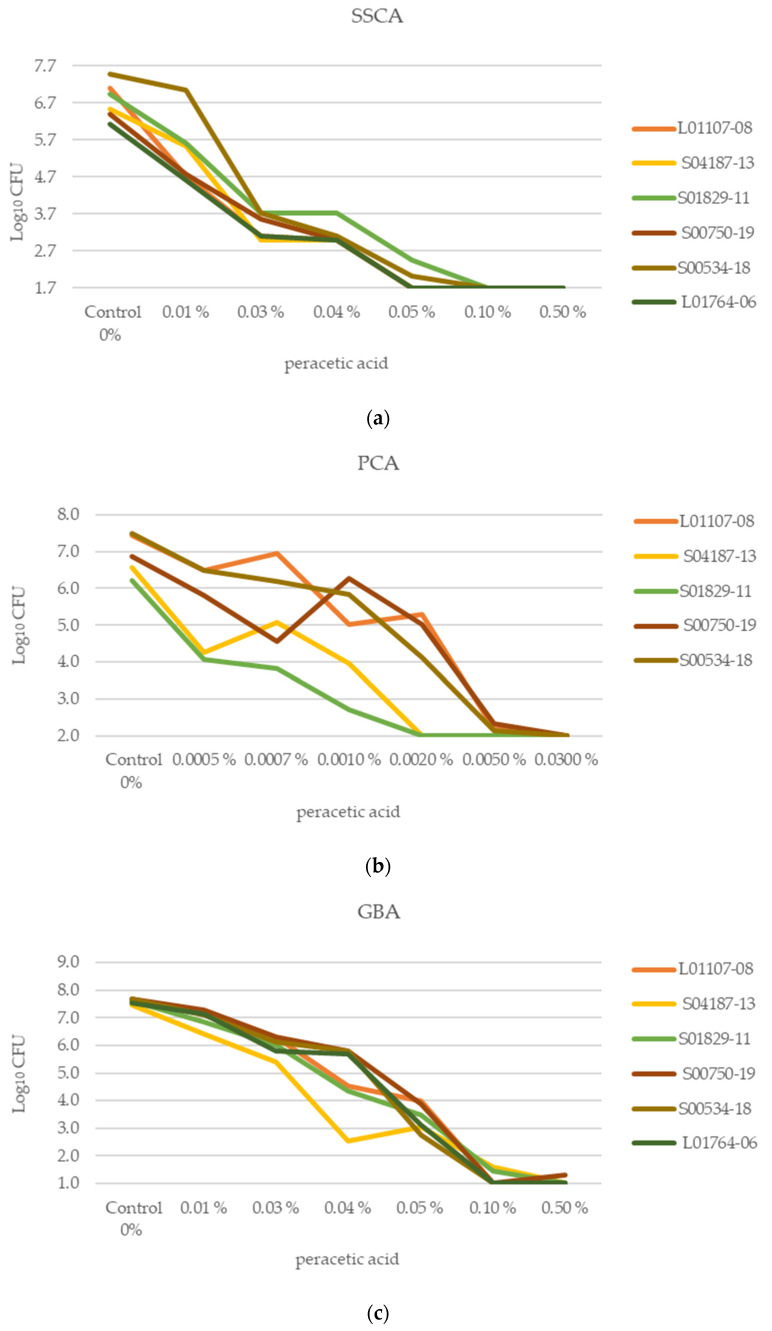
(**a**–**c**). Log_10_ CFU of separate strains following treatment of biofilms with peracetic acid of various concentrations in (**a**) Stainless steel coupon assay (SSCA), with limit of detection (LOD) = 1.7 log_10_ CFU, (**b**) PVC coupon assay (PCA) with LOD = 2 log_10_ CFU and (**c**) glass bead assay (GBA) with LOD = 1 log_10_ CFU on wild-type strains of 3 experiments with 2 (SSCA and PCA) or 3 (GBA) technical replicates each. Note that the data are non-continuous. Standard deviation (SD) can be found in S3.

**Figure 3:**
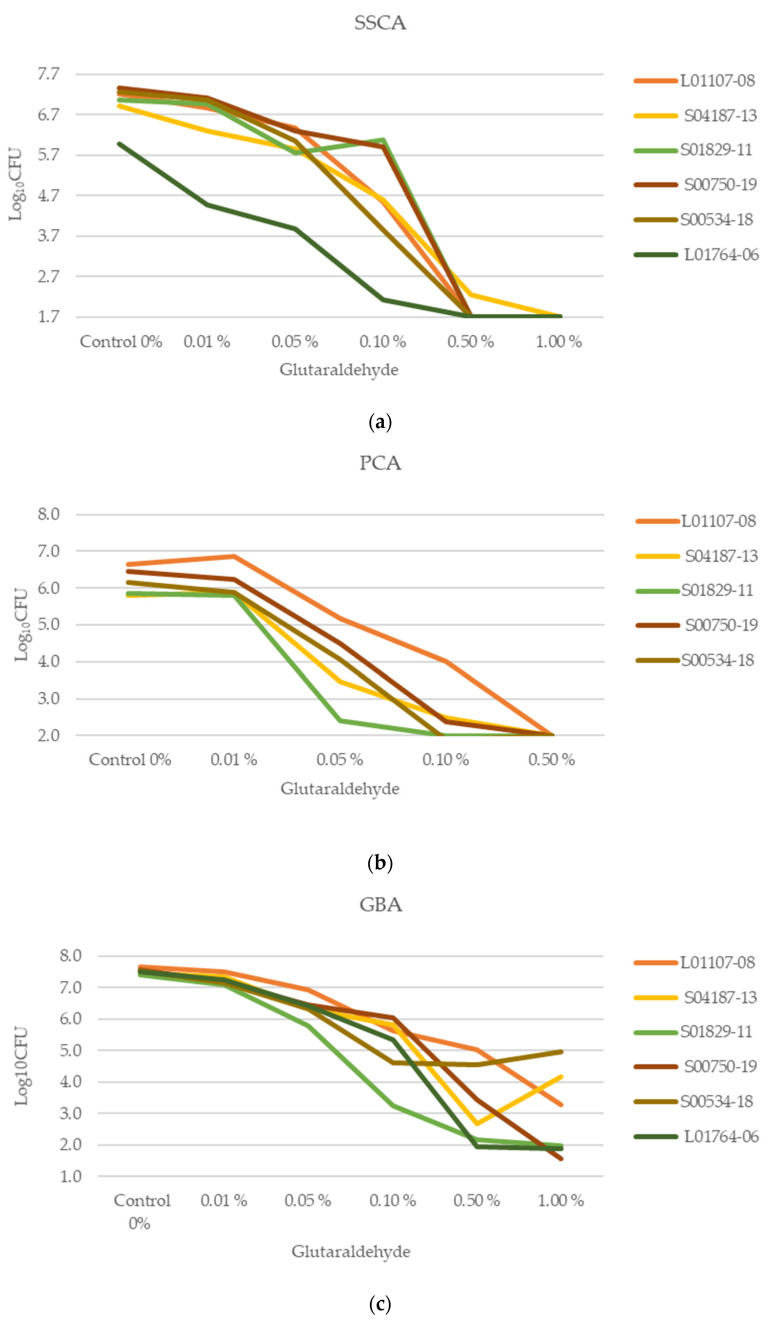
(**a**–**c**). Log_10_ CFU of separate strains after treatment of biofilms with glutaraldehyde of various concentrations in (**a**) Stainless steel coupon assay (SSCA), with limit of detection (LOD) = 1.7 log_10_ CFU, (**b**) PVC coupon assay (PCA) with LOD = 2 log_10_ CFU and (**c**) glass bead assay (GBA) with LOD = 1 log_10_ CFU on wild-type strains of 3 experiments with 2 (SSCA and PCA) or 3 (GBA) technical replicates each. Note that the data are non-continuous. Standard deviation (SD) can be found in S3.

To detect a reduction ≥4 log_10_ CFU for a strain, the mean log_10_ CFU of the untreated strain had to be at least 4 log_10_ CFU above the limit of detection (LOD) of the assay used, resulting in a minimum of 5.7 log_10_, 5.0 log_10_, and 6.0 log_10_ CFU for SSCA, PCA, and GBA, respectively. Strain L01765-06, which also showed the weakest biofilm formation in the MTP assay, was, therefore, excluded for the PCA, as it did not reach consistently high cell numbers_._ Figure 2a–c show the effect of peracetic acid on each strain within each assay. Figure 3a–c show the effect of glutaraldehyde on each strain within each assay. The mean log_10_ CFU and the standard deviation (SD) for each strain in all three assays are included in the Supplementary Materials (Table S3).

#### 2.4.2. Mean log_10_ CFU Reduction after Disinfection

The requirement for efficient disinfection was considered to be met when the mean log_10_ reduction in the selected test strains was ≥4 log_10,_ as shown in Table 4, Table 5 and Table 6. The concentrations of peracetic acid that gave the required reduction were 0.05% in SSCA and GBA (Table 4), and in PCA, the concentration was 0.005% (Table 5). For glutaraldehyde, the concentration that gave the required reduction was 0.50% in all three assays (Table 6).

## 3. Discussion

Using wild-type *Salmonella* strains from the pig industry, this study showed that when looking at pooled results for all strains, the minimum effective concentrations of peracetic acid needed to obtain the required mean reduction (≥4 log_10_CFU) of the strains in biofilm were 0.05% in the SSCA and GBA and 0.005% in the PCA. For glutaraldehyde, the concentrations were ≥0.5% for all three assays. Although the wild-type strains tested showed variable responses to the disinfectants, all the individual strains displayed the required log_10_CFU reduction at these concentrations when tested in SSCA and PCA, and 67% of the strains when tested in GBA. Consequently, we believe that the results from testing this group of representative strains provide valuable information on the effect of these disinfectants on biofilm-residing salmonella in the pig industry. As there are no official standards for testing the effect of disinfectants on *Salmonella enterica* subspecies *enterica* in biofilms, a ≥4 log_10_CFU reduction was chosen based on the standard EN 13697:2015+A1:2019 [20], which is a quantitative non-porous surface test for the evaluation of bactericidal and/or fungicidal activity of chemical disinfectants used in food, industrial, domestic, and institutional areas. Moreover, our recently published study demonstrated that disinfectant concentrations, which were evaluated using standardized protocols for the disinfection of cell suspensions (EN 1656), are not high enough to achieve a ≥4 log_10_CFU reduction when tested against biofilms. In this study, on the *S.* Typhimurium reference strain ATCC 14028, the concentrations of the same disinfectants needed to give a ≥5 log_10_ reduction, according to EN 1656, were 0.03% with glutaraldehyde and 0.002% with peracetic acid [24,29]. This shows that there is a necessity for significantly higher concentrations of disinfectants when biofilms are a concern and emphasizes the importance of also testing bacteria in biofilms when evaluating disinfectants on wild-type strains [30]. When performing laboratory studies on planktonic bacteria, the experiments are easier to standardize with regard to growth conditions, cultivation, and the concentration of bacteria in the test suspension. In addition, individual cells within batch cultures are more homogenous concerning gene expression and stress tolerance. Biofilms are more heterogeneous cell communities, as biofilm formation and extracellular matrix composition will vary according to growth conditions. They may be affected by several factors, such as surface material for biofilm adhesion, shear stress, as well as nutrient and oxygen supply in the surrounding media [31,32,33].

Furthermore, bacteria in biofilms are more tolerant to biocides compared to planktonic cells due to several mechanisms/properties of the biofilm [28]. One of these is the physical and chemical barrier of the exopolysaccharide matrix. Others include the development of resistance mechanisms, altering into another physiological state (such as dormant cells), and changes in the microenvironment [34,35,36]. These mechanisms collectively make the bacteria tolerant to higher concentrations or exposed to sub-inhibitory concentrations of the disinfectants [37]. As the biofilm ages (which is often the case in environmental biofilms), the cell density increases, and the problem is exacerbated [21]. A study by Vestby et al. in 2009 showed that the *Salmonella* serovars, which persisted more readily under factory conditions, were better biofilm producers [38]. In the present study, when looking at the pooled results for all strains, the mean effective glutaraldehyde and peracetic acid concentrations necessary for the required reduction varied less than could be expected between the three different assays used. For glutaraldehyde, the effective concentration was the same in all three assays. Also, for peracetic acid, the effective concentration was the same when using SSCA and GBA but notably higher than that found in the PCA. It should be noted that the conditions of SSCA and PCA are more similar than GBA, as they are both coupon-based static methods, as opposed to glass beads and non-static conditions in the GBA. However, the coupons in the PCA assay are smaller than in SSCA and not removed by scraping as in the latter. Some parameters between the three assays vary, and the amount or composition of the biofilm created differs. Both factors can be expected to influence the efficacy of disinfectants. There was a difference in the biofilm environments of the assays, which may influence both the structure and the components of the biofilm and explain the discrepancy in the PCA results. A marked difference has previously been reported in the morphology of the water-facing and air-facing biofilm surfaces of *Salmonella* biofilm pellicles in the liquid–air interface [39]. It was shown that the air-facing biofilm contains an exocellular integument with a homogenous surface, while the water-facing surface was clustered and with less integument [39]. The SSCA and PCA were strictly static, and the biofilm in the liquid–air interphase was used. However, the GBA is not static, and the majority of the biofilm was formed below the liquid–air interphase but in an oxygen-rich environment due to shaking. In other bacteria-producing biofilms in the liquid–air interface, it has been seen that biofilm development was increased when shear forces were applied [40]. The GBA may resemble areas in the farm environment where bacteria are exposed to shear forces, such as in waterpipes, whereas the SSCA and PCA may resemble other parts of the farm environment, such as drinking/feed troughs or cracks in surfaces, which are hard to clean. Both are areas where biofilms are prone to develop. As such, one might anticipate a hardship in standardizing assays for measuring the effect of disinfectants on bacteria in biofilms. However, this study shows that this may be more accessible than expected.

In the current study, we also looked at the correlation between biofilm production in the MTP assay and the three assays used in the disinfectant testing. In the MTP assay, biofilm production was measured using CV, which stains the biofilm matrix in addition to live and dead cells [41]. However, in the other assays, we assessed the surviving cells by counting the CFUs. Therefore, the biomass formed may vary, even though the number of surviving CFUs is similar. Nonetheless, positive correlations were observed between the MTP assay results and CFU counts in the SSCA and PCA. This correlation was not seen in the GBA. This may be due to the similar conditions in the MTP assay to SSCA and PCA (static incubation, biofilm in the liquid–air interface). In addition, more biofilm was formed in the GBA than in the other assays, and even the L01764-06 strain possessing a low-biofilm-forming capacity in the MTP assay developed substantial biofilms (with respect to cell numbers) in the GBA approach.

In spite of the limitation that the BDAR morphotype was only represented with one strain and could not be compared as such, there was an insinuation with the SSCA that peracetic acid had the same effect on all strains, independent of colony morphology, while glutaraldehyde was more effective against BDAR compared to the RDAR morphotype. However, this must be considered an indication and not as a fact, and more studies are necessary. In the MTP assay screening, the *S*. Typhimurium strains produced more biofilm than the *S*. Derby strains. There was also a higher percentage of *S*. Typhimurium with the RDAR morphotype compared to *S.* Derby. Previous studies on *E. coli* have shown that the age of the biofilm and the morphology are important factors [21,32] in biofilm sustainability. It has been shown that the combination of curli and cellulose in the matrix forms a tissue-like mesh around the cells, thus protecting them from harsh environmental conditions [42,43], which may form a barrier for disinfectants. The strains with biofilm of RDAR morphology in *E. coli* are known to have a higher mean biofilm production than BDAR strains [32], withstand the treatment with disinfectants, and, therefore, to better survive in the environment [21]. They have also been found to display a larger CFU reduction after treatment of disinfectants on 2-day-old biofilms of BDAR strains compared to 5-day-old biofilms [21] for *E. coli*. In a previous study testing disinfectants on *S.* serovar *Agona* biofilms, this interrelationship was not found [38]. However, those BDAR strains were also strong biofilm formers in the MTP assay. This may also influence the biofilm formation on coupons and beads and, thereby, the disinfection. Nevertheless, RDAR is the most common morphotype in wild-type *Salmonella* and *E. coli* [15,17]. In fact, most *S. enterica* subspecies *enterica* wild-type strains produce curli fimbriae in the matrix, and 40–100% produce cellulose as well, depending on the serovar and the source of isolation [18,38,44]. In addition to this, the RDAR morphology is also known to increase as the temperature is decreased [45], such as in production facilities. Accordingly, it may be possible that *S*. Derby does not survive as well in the environment as *S*. Typhimurium. This may also be the reason that *S*. Derby is found in a higher percentage as BDAR or SAW morphotypes, the latter producing neither curli nor cellulose. Therefore, when evaluating the effect of disinfectants, it is necessary to study wild-type strains and strong biofilm producers to better reflect the most resilient conditions in the pig-housing environments. It is imperative, when assessing the effect of disinfectants, to take into account the variation between strains. Some strains have a lower biofilm production, and some are more resilient, with a high biofilm production. As it is not beneficial to use a less-than-effective concentration or to overuse a disinfectant (this will adversely affect the surrounding environment), we assessed the mean in this study.

Glutaraldehyde is known as a potent disinfectant, and its effect is reliant on the aldehyde group. This alkylates the sulfhydryl, hydroxyl, carboxyl, and amino groups of bacterial proteins, instigating protein coagulation and cell death [46]. Peracetic acid action is relative to the production of reactive free radicals that oxidize bacteria and biofilms. This is caused by the hydroxyl radical and reactive peroxidase primarily causing the biocide activity [47]. The difference in mode of action of the two disinfectants may explain the slight differences in the effect of glutaraldehyde for RDAR and BDAR morphotypes using the SSCA. Curli fimbriae are fibrous surface proteins and are suggested to be of critical position in adhesion to surfaces [48], while cellulose has been known to have a protective effect and is able to withstand strong acids and alkalis [17,18], which may be the reason for the implication seen in the present study.

## 4. Materials and Methods

### 4.1. Bacterial Strains and Media

*S. enterica* wild-type strains serovars *Salmonella* (*S.*) Derby (*n* = 15) and *S.* Typhimurium (STM) (*n* = 36), including monophasic variants (mSTM) (*n* = 13), with phage types DT193 and U288 OR U302 from the pig industry, were selected from the Animal and Plant Health Agency (APHA) *Salmonella* strain collection [49,50,51]. These strains were previously sourced from pig faeces collected from finisher pigs or sows/gilts on UK indoor pig finishing, fattening, or breeding farms and were collected between 2006 and 2019. The strains were further screened for biofilm production and morphology, as described below (Tables S1 and S2). Based on these results, six representative strains were selected (Table 3). The strains included in the study (Table 3) were stored at −80 °C, before recovery on 5% sheep blood agar (SBA) in tryptic soy broth (TSB) or Luria Bertani (LB) medium according to each assay (see below). Recovery medium and plates for enumeration of colony-forming units (CFUs) were incubated at 37 ± 1 °C overnight under aerobic conditions.

### 4.2. Biofilm Production on Polystyrene (Microtiter Plate Assay)

The strains were screened for their biofilm-forming capability using incubation in Luria–Bertani (LB) broth without NaCl (bacto tryptone (10 g/L), yeast extract (5 g/L), and distilled water) for 48 h at 20 ± 1 °C in the Crystal Violet (CV) microtiter plate (MTP) assay [12]. Each strain was assessed in triplicate, and optical density (OD_600_) of 1% CV bound by attached biofilms was measured following dissolving with 95% ethanol: 5% reverse osmosis water (*v*/*v*). For each experiment the median OD_600_ for each strain was calculated and the median blank OD_600_ subtracted. Further, the mean OD_600_ between two experiments was calculated (Table 3 and Tables S1 and S2).

### 4.3. Biofilm Morphotyping

*Salmonella* colony morphology was assessed according to [32,52], following incubation for 4 days at 20 ± 1 °C on LB agar plates without NaCl supplemented with Congo Red (0.04 g/L) and Coomassie Brilliant blue (0.02 g/L) (CRCB) (Sigma-Aldrich, St Louis, MO, USA) [53]. In brief, 1 µL of overnight cultures was spot-inoculated on CRCB plates. After incubation, they were examined visually and categorized as RDAR, expressing curli fimbriae and cellulose, BDAR, expressing fimbriae but not cellulose, or SAW, expressing neither cellulose nor fimbriae (Table 3 and Tables S1 and S2). Experiments were repeated at least three times.

### 4.4. Disinfectant Testing

#### 4.4.1. Study Design

Glutaraldehyde (Protectol GA 50, BASF, Ludwigshafen, Germany) and peracetic acid (Lerasept ^®^ Spezial, Stockmeier Chemie, Bielefeld, Germany) were chosen according to their relevance to the pig industry. They were diluted to the desired concentrations in hard water (HW), which was prepared according to DIN EN 1656:2019-10 [29]. The controls were treated with HW instead of disinfectant. The glutaraldehyde and peracetic acid concentrations tested via each assay within the study are described in Table 7. The exposure time for glutaraldehyde was 30 min and 10 min for peracetic acid. Treatment with glutaraldehyde was followed by neutralizing with a composition of 10 g/L Tween 80 and 20 g/L Glycine in 0.25 M phosphate buffer (pH 7). Treatment with peracetic acid was followed by neutralizing with a composition of 1.65% sodium sulfite in 0.1 M phosphate buffer (16.5 g/l Na_2_SO_3_ in 0.1M phosphate buffer (KH_2_PO_4_ + Na_2_HPO_4_ × 2H_2_O)). Both disinfectants were neutralized for at least five minutes. Neutralizer toxicity and disinfectant neutralization validation checks were performed, as described in Richter/Konrat et al. 2023 [24].

#### 4.4.2. Criteria for Successful Disinfection

Efficient disinfection was defined as a log_10_CFU reduction ≥4, according to the requirements in the European surface test (2015) (EN 13697:2015+A1:2019) [20], the quantitative non-porous surface test used in food, industrial, domestic, and institutional areas. When testing all strains included, we considered this requirement met when the mean log_10_CFU reduction in the tested strains was ≥4.

#### 4.4.3. Stainless-Steel Coupon Assay (SSCA)

The method was performed according to Richter/Konrat et al. 2023 [24] with some modifications. To create a working culture in broth, bacterial cultures on blood agar were transferred to 5 mL LB, and the OD was measured and adjusted to 1 McFarland. Then, 0.5 mL of each bacterial suspension was added to 10 mL of LB without NaCl together with an autoclaved stainless-steel coupon of 75 × 24 × 1 mm (stainless steel AISI304, 2B Olaf Johansens Eftf. A/S, Oslo, Norway) and incubated at 20 ± 1 °C for 48 h. Thereafter, the coupon was rinsed 3× in 40 mL sterile saline and transferred to a tube with 10 mL of disinfectant for the applicable exposure time (see above). The coupon was moved to a tube with the appropriate neutralization agent before it was rinsed 3× again and added to a tube containing 20–30 2 mm glass beads (soda lime glass, Merck, Darmstadt, Germany) and 5 mL saline. Here, visible biofilm was scraped off both sides of the coupon by using a cell scraper with a blade of 1.8 cm (BD Falcon, Bedford, MA, USA). The coupon was discarded before the tube was vortexed for one minute. An aliquot of 0.2 mL from each tube was added to wells in a microtiter plate. Serial dilutions were performed before plating 5 μL on blood agar to determine the total viable CFU count per coupon. To confirm the absence of growth at selected concentrations, 100 µL of undiluted solution was plated. All experiments were performed in duplicate and conducted three times. Level of detection (LOD) was set to 1.7 log_10_ when no viable bacteria were observed. See reference [24] for more detail on calculation of LOD.

#### 4.4.4. Polyvinylchloride (PVC) Coupon Assay (PCA)

The method was performed according to Richter/Konrat et al. 2023 [24]. *Salmonella* strains were inoculated into LB without NaCl and the bacterial suspension adjusted to 1 McFarland (~3 × 10^8^ CFU/mL), using a turbidity suspension meter. To each well of a 12-well microtiterplate, 1.5 mL of this bacterial suspension was added, with one well containing sterile broth. One sterile polyvinyl carbonate (PVC) coupon (satin finish, 10 × 20 × 1.5 mm, Hygienic Plastics Supplies Ltd., Lancashire, UK) was placed into each well so that only the bottom half of the longest side of the coupon was submerged in the bacterial suspension. Microtiter plates underwent static aerobic incubation for 48 h at 20 ± 1 °C. After incubation, each coupon was washed three times, with light agitation, in three consecutive 9 mL sterile saline solutions and left to dry at room temperature. Coupons were submerged in either 10 mL disinfectant or 10 mL hard water for the specified exposure time (see above) before being transferred to 10 mL of the appropriate neutralizer broth. After a minimum of 5 min neutralization time, coupons were transferred to 5 mL sterile saline with 30 glass beads (5 mm soda lime glass beads (Z265942; Sigma-Aldrich, St. Louis, MI, USA)) and shaken at the lowest speed on a vortex mixer for 2 min, before a further 5 mL of saline was added. Serial dilutions were performed and 100 µL spread-plated on 5% sheep blood agar plates to allow for enumeration of viable CFU count per coupon. Experiments were performed with two technical replicates and three biological replicates. LOD was set to 2 log_10_ when no viable bacteria were observed. See reference [24] for more detail on calculation of LOD.

#### 4.4.5. Glass Bead Assay (GBA)

The method was performed according to Richter/Konrat et al. 2023 [24]. Biofilms were cultivated on 4 mm porous glass beads (Sinterglas Pellets, ROBU Glasfilter-Geräte GmbH, Hattert, Germany) in 24-well microtiterplates (one bead per well). Each well was inoculated with 1 mL LB without NaCl containing 10^5^ CFU/mL *S.* Typhimurium or *S.* Derby. Plates were incubated on an orbital shaker at 100 rpm at 20 ± 1 °C for 48 h for biofilm cultivation. Thereafter, each bead was carefully dipped in 2 mL sterile H_2_O and placed in a 2 mL microcentrifuge tube containing 0.2 mL disinfectant. After incubation for the defined exposure time, 1.8 mL of the neutralizing agent was added to each microcentrifuge tube. Subsequently, bacteria were sonicated in an ultrasonic bath (BactoSonic^®^, Bandelin, Berlin, Germany) at 40 kHz for 10 min using 200 W_eff_ to detach the biofilm from the bead surface and quantified via serial dilution. All dilution steps were performed in neutralizer. A total of 5 µL of individual dilutions was spotted on Tryptic Soya broth (TSB) agar plates. In addition, 1 mL of undiluted solutions and the first dilution were also plated on TSB agar. Results are presented in CFU/mL. All experiments were performed three times with three technical replicates each. The LOD was set to 1 log_10_ when no viable bacteria were observed. See reference [24] for more detail on calculation of LOD.

### 4.5. Statistics

All statistical analyses were performed using Excel vs. 2016 (Microsoft, Redmond, WA, USA). Means of groups of wild-type strains were compared using a two-tailed Student’s *t*-test, and *p* ≤ 0.05 was considered statistically significant. In the disinfection experiment, the log_10_CFU reduction for each strain was calculated by taking the mean of the controls in the three experiments and subtracting the log_10_CFU after treatment with disinfectants. Further, an average of the log_10_CFU reduction in all strains studied was calculated.

## 5. Conclusions

In conclusion, this study shows that biofilms should be included when designing disinfection strategies. Testing the disinfection of planktonic bacteria alone may result in a recommendation of ineffective concentrations. Furthermore, it highlights the intricacy and complexity of testing disinfectants on biofilms. Although some differences were seen between the assays used, all three assays identified the same effective concentration of glutaraldehyde to reach the required mean reduction in the group of representative wild-type *Salmonella* strains in the biofilm, while PCA required a lower concentration than the other two assays to reach the same mean reduction with peracetic acid. This study was performed under ‘optimal’ lab conditions, and the results may differ when disinfectant products are used on farms due to factors, such as temperature, organic matter (due to insufficient cleaning), and other chemicals present in commercial disinfectants. Other factors that may influence the effect include the composition of the biofilm being treated and the surface material that it adheres to. Nevertheless, our findings suggest that when used in sufficient concentrations, glutaraldehyde- and peracetic-acid-based disinfectants may generally be suitable for controlling *Salmonella* biofilms on pig farms, but more work is needed to determine their efficacy in real-world settings.

## Figures and Tables

**Table 1:** Mean optical density (OD_600)_) ± standard deviation (SD) in the MTP assay of different serovars.

	No	Mean OD_600_ ± SD
*S*. Typhimurium	35	0.851 ± 0.189 ^A^
*S*. Derby	15	0.189 ± 0.298 ^B^

No = number of strains. Different letters ^A,B^ indicate significantly different means (*p* < 0.001).

**Table 2:** Mean optical density (OD_600)_) ± standard deviation (SD) in the MTP assay of different morphotypes.

Morphotype	No	Mean OD_600_ ± SD	% of *S*T	% of *S*D
RDAR	31	0.892 ± 0.294 ^A^	77.1	26.7
BDAR	6	0.507 ± 0.327 ^B^	17.1	0.0
SAW	13	0.149 ± 0.222 ^B^	5.7	73.3

No = number of strains. RDAR = red, dry and rough colonies. BDAR = brown, dry and rough colonies. SAW = smooth and white colonies. Different letters ^A,B^ indicate significantly different means (*p* < 0.001). *S*T = *S*. Typhimurium (including monophasic variants). *S*D = *S*. Derby.

**Table 3:** The strains selected for the disinfection experiments.

Strain	Serovar	Production Stage ^1^	OD_600nm_ in MTP Assay ^2^	Morphology ^3^	Phage Type
S04187-13	*S.* Typhimurium	Finisher	1.21	RDAR	U288
L01107-08	*S.* Typhimurium	Finisher	1.10	RDAR	DT193
S00750-19	Monophasic *S.* Typhimurium	Finisher	0.97	RDAR	DT193
S00534-18	Monophasic *S.* Typhimurium	Sow/gilt	0.92	RDAR	DT193
S01829-11	*S.* Derby	Finisher	0.97	RDAR	n/a
L01764-06	*S.* Typhimurium	Finisher	0.18	BDAR	U302

^1^ production stage of the pigs at point of sample collection level ^2^ biofilm formation in MTP assay ^3^ morphology on Congo Red Coomassie blue (0.040 g/L Congo Red and 0.02 g/L Coomassie Blue) (CRCB) plates.

**Table 4:** Mean log_10_ CFU ± confidence interval (CI)95% reduction after treatment with peracetic acid on biofilms of six wild-type strains by using the SSCA and GBA.

		Controls Mean Total log_10_CFU	Mean log_10_CFU Reduction ± CI 95% at Each Peracetic Acid Concentration					
Assay	LOD ^a^ log_10_CFU		0.01%	0.03%	0.04%	0.05%	0.10%	0.50%
SSCA	1.7	6.76 ± 0.52	1.38 ± 0.67	3.40 ± 0.46	3.63 ± 0.53	4.88 ± 0.47	5.06 ± 0.53	5.06 ± 0.53
GBA	1	7.60 ± 0.10	0.59 ± 0.28	1.61 ± 0.28	2.83 ± 1.27	4.23 ± 0.48	6.43 ± 0.3 6	6.55 ± 0.11

Grey background = required reduction (≥4 log_10_) met. ^a^ Limit of detection (LOD). SSCA = Stainless steel coupon assay. GBA = Glass bead assay.

**Table 5:** Mean log_10_CFU ± CI 95% reduction after treatment with peracetic acid on biofilms of five wild-types strains by using the PCA.

		Controls Mean Total log_10_CFU	Mean log_10_CFU Reduction ± CI at each Peracetic Acid Concentration					
Assay	LOD ^a^ log_10_CFU		0.0005%	0.0007%	0.001%	0.002%	0.005%	0.03%
PCA	2	6.92 ±0.59	1.49 ±0.83	1.61 ±0.98	2.16 ±1.35	3.24 ±1.50	4.79 ± 0.61	4.92 ± 0.69

Grey background = required reduction (≥4 log_10_) met. ^a^ Limit of detection (LOD). PCA = PVC coupon assay.

**Table 6:** Mean logCFU ± CI 95% reduction after treatment with glutaraldehyde on biofilms of six wild-type strains by using the SSCA and GBA, and five using the PCA.

		Controls Mean Total log_10_CFU	Mean log_10_CFU Reduction ± CI 95% at Each Glutaraldehyde Concentration				
Assay	LOD ^a^ log_10_CFU		0.01%	0.05%	0.10%	0.50%	1.00%
SSCA	1.7	6.97 ±0.54	0.51 ± 0.54	1.27 ± 0.47	2.45 ± 1.15	5.18 ± 0.59	5.27 ± 0.53
PCA	2	6.18 ±0.39	0.05 ± 0.25	2.26 ± 0.92	3.63 ± 0.81	4.18 ± 0.46	^b^
GBA	1	7.51 ±0.13	0.26 ± 0.12	1.13 ± 0.29	2.40 ± 1.04	4.21 ± 1.26	4.54 ± 1.44

Grey background = required reduction (≥4 log_10_) met ^a^ Limit of detection (LOD) ^b^ Not included in PCA. SSCA = Stainless steel coupon assay. PCA = PVC coupon assay. GBA = Glass bead assay.

**Table 7:** The concentrations tested for glutaraldehyde and peracetic acid for each assay.

	Glutaraldehyde					Peracetic Acid					
SSCA	0.01%	0.05%	0.1%	0.5%	1%	0.01%	0.03%	0.04%	0.05%	0.1%	0.5%
GBA	0.01%	0.05%	0.1%	0.5%	1%	0.01%	0.03%	0.04%	0.05%	0.1%	0.5%
PCA	0.01%	0.05%	0.1%	0.5%	-	0.0005%	0.0007%	0.001%	0.002%	0.005%	0.03%

SSCA = Stainless steel coupon assay. GBA = glass bead assay. PCA = PVC coupon assay.

## Data Availability

The data presented in this study are available on request from the corresponding authors.

## Supplementary Materials

The following supporting information can be downloaded at: https://www.mdpi.com/article/10.3390/antibiotics12071189/s1, Table S1. Biofilm classification and colony morphotype *S*. Derby. Table S2. Biofilm classification and colony morphotype *S*. Typhimurium. Table S3. The mean log_10_ CFU for each strain in each assay and for both disinfectants with standard deviation.
