# Supplementary material for: Evaluation of Disinfectant Efficacy against Biofilm-Residing Wild-Type Salmonella from the Porcine Industry
Source: Antibiotics (Basel). 2023 Jul 14;12(7):1189. doi: 10.3390/antibiotics12071189 (PMC10376135; doi:10.3390/antibiotics12071189)
Supplement: Supplementary file 1 [file antibiotics-12-01189-s001.zip › antibiotics-2505623-supplementary.pdf]

Supplementary Materials

Table S1. Biofilm production in the microtiter plate assay and colony morphotype by wild-type *S*. Derby isolates from the pig industry

Isolate	Production stage	Year isolated	OD_{600} in MTP[a]	Morphotype[b]
**L00665-06**	Finisher	2006	0.037	saw
**L00840-07**	Finisher	2007	0.041	saw
**L01888-06**	Finisher	2006	0.043	saw
**S05998-15**	Sow/gilt	2015	0.048	saw
**S01708-07**	Finisher	2007	0.049	saw
**L00074-07**	Finisher	2007	0.050	saw
**S04186-13**	Finisher	2013	0.052	rdar
**S01160-06**	Finisher	2006	0.053	saw
**S04854-11**	Sow/gilt	2011	0.056	saw
**L00834-07**	Finisher	2007	0.069	saw
**L01030-08**	Finisher	2008	0.073	saw
**S01523-07**	Finisher	2007	0.077	saw
**L01583-06**	Finisher	2006	0.274	rdar

**L00870-08**	Finisher	2008	0.800	rdar
**S01829-11**[c]	Finisher	2011	0.971	rdar

[a] Optical density (OD) in microtiter plates. [b] saw = smooth and white colonies. rdar = red. dry and rough colonies. [c] Included in disinfection studies

Table S2. Biofilm production in the microtiter plate assay and colony morphotype of wild-type *S*. Typhimurium (including monophasic variants) isolates from the pig industry

Isolate	Serovar	Phage Type	Production stage	Year isolated	OD_{600} in MTP[a]	Colony morphology[b]
**S00391-19**	ST	DT193	Sow/gilt	2018	0.038	bdar
**S02329-19**	mST	DT193	Finisher	2018	0.167	rdar
**L01764-06**[c]	ST	U302	Finisher	2006	0.182	bdar
**S01706-07**	ST	U288	Finisher	2007	0.280	rdar
**L00482-08**	ST	DT193	Finisher	2008	0.466	rdar
**L00762-07**	ST	U288	Finisher	2007	0.546	bdar
**S00076-11**	mST	DT193	Sow/gilt	2010	0.574	saw
**S01125-09**	mST	DT193	Finisher	2009	0.612	rdar
**S05813-15**	mST	DT193	Sow/gilt	2015	0.669	saw
**S01872-07**	ST	U288	Finisher	2007	0.677	bdar
**S02075-07**	ST	U288	Finisher	2007	0.731	bdar
**L00836-07**	ST	U288	Finisher	2007	0.740	rdar
**S02190-12**	ST	DT193	Finisher	2012	0.866	bdar
**L01105-18**	mST	DT193	Sow/gilt	2018	0.894	rdar
**S01671-11**	ST	U288	Finisher	2011	0.911	rdar
**S00534-18**[c]	mST	DT193	Sow/gilt	2017	0.918	rdar
**L00381-07**	ST	U288	Sow/gilt	2007	0.933	rdar
**S05778-18**	mST	DT193	Finisher	2018	0.952	rdar
**S00522-08**	ST	DT193	Finisher	2008	0.956	rdar

**S00750-19**[c]	mST	DT193	Finisher	2019	0.968	rdar
**S04907-12**	mST	DT193	Finisher	2012	0.989	rdar
**L01033-08**	ST	DT193	Finisher	2008	0.990	rdar
**L00064-16**	mST	DT193	Finisher	2016	1.017	rdar
**S03694-19**	mST	DT193	Sow/gilt	2018	1.025	rdar
**S01524-07**	ST	U288	Finisher	2007	1.026	rdar
**L00872-08**	ST	DT193	Sow/gilt	2008	1.031	rdar
**L00833-07**	ST	U302	Finisher	2007	1.047	rdar
**S00085-07**	ST	U302	Finisher	2006	1.065	rdar
**S05947-07**	ST	U288	Sow/gilt	2007	1.094	rdar
**S00479-12**	mST	DT193	Finisher	2012	1.096	rdar
**L01107-08**[c]	ST	DT193	Finisher	2008	1.097	rdar
**S04541-12**	ST	U288	Finisher	2012	1.104	rdar
**S05574-08**	mST	DT193	Finisher	2008	1.142	rdar
**L01196-08**	ST	DT193	Finisher	2008	1.204	rdar
**S04187-13**[c]	ST	U288	Finisher	2013	1.213	rdar

[a] Optical density (OD) in microtiter plates. [b] saw = smooth and white colonies. bdar = brown. dry and rough colonies. rdar = red. dry and rough colonies.
[c] Included in disinfection studies

Table S3 The mean achievable $\log_{10}$ CFU for each strain in each assay and for both disinfectants with standard deviation (SD)

		L01107-08		S04187-13		S01829-11		S00750-19		S00534-18		L01764-06	
		$\log_{10}$CFU	± SD	$\log_{10}$CFU	± SD	$\log_{10}$CFU	± SD	$\log_{10}$CFU	± SD	$\log_{10}$CFU	± SD	$\log_{10}$CFU	± SD
						**Peracetic acid**							
SSCA	Control 0%	7.09	0.45	6.54	0.12	6.93	0.35	6.39	0.16	7.49	0.18	6.13	0.04
	0.01 %	4.72	0.89	5.53	1.28	5.61	1.11	4.78	0.52	7.04	0.33	4.61	0.81
	0.03 %	3.10	0.10	3.00	0.00	3.71	0.43	3.55	0.55	3.73	0.42	3.10	0.10
	0.04 %	3.00	0.00	3.00	0.00	3.72	0.72	3.00	0.00	3.10	0.10	3.00	0.00
	0.05 %	1.70	0.00	1.70	0.00	2.45	0.75	1.70	0.00	2.02	0.32	1.70	0.00
	0.10 %	1.70	0.00	1.70	0.00	1.70	0.00	1.70	0.00	1.70	0.00	1.70	0.00
	0.50 %	1.70	0.00	1.70	0.00	1.70	0.00	1.70	0.00	1.70	0.00	1.70	0.00
PCA	Control 0%	7.45	0.23	6.57	0.68	6.22	0.77	6.88	0.26	7.50	0.54	5.50	0.33
	0.0005%	6.50	0.91	4.27	2.50	4.08	0.78	5.82	2.23	6.48	0.46	a	a
	0.0007%	6.95	0.49	5.08	1.04	3.82	1.85	4.55	2.39	6.18	1.06	a	a
	0.001%	5.02	3.28	3.95	2.15	2.72	1.24	6.28	0.83	5.83	1.43	a	a
	0.002%	5.28	0.67	2.00	0.00	2.00	0.00	5.03	0.85	4.12	1.08	a	a
	0.005%	2.20	1.31	2.00	0.00	2.00	0.00	2.32	0.35	2.15	0.26	a	a
	0.03%	2.00	0.00	2.00	0.00	2.00	0.00	2.00	0.00	2.00	0.00	a	a
GBA	Control 0%	7.65	0.13	7.45	0.18	7.60	0.13	7.70	0.30	7.67	0.13	7.53	0.03
	0.01 %	7.25	0.26	6.40	0.05	6.87	0.16	7.30	0.10	7.12	0.28	7.15	0.09
	0.03 %	6.32	0.16	5.38	0.39	6.02	0.33	6.32	0.08	6.13	0.20	5.80	0.13
	0.04 %	4.52	1.19	2.53	1.53	4.33	0.85	5.78	0.60	5.78	0.81	5.68	0.38
	0.05 %	4.00	0.97	3.03	1.24	3.47	1.45	3.87	1.71	2.75	2.65	3.12	0.48
	0.10 %	1.00	0.00	1.60	0.52	1.45	0.78	1.00	0.00	1.00	0.00	1.00	0.00
	0.50 %	1.00	0.00	1.00	0.00	1.00	0.00	1.30	0.52	1.00	0.00	1.00	0.00
						**Glutaraldehyde**							
SSCA	Control 0%	7.21	0.27	6.92	0.05	7.07	0.05	7.36	0.17	7.27	0.22	5.98	0.02

	0.01 %	6.86	0.21	6.30	0.25	6.97	0.11	7.10	0.05	7.06	0.23	4.98	0.48
	0.05 %	6.37	0.16	5.85	0.44	5.76	0.39	6.31	0.23	6.05	0.50	4.09	0.02
	0.10 %	4.55	0.68	4.60	1.45	6.07	0.40	5.91	0.07	3.86	1.23	2.13	0.43
	0.50 %	1.70	0.00	2.26	0.56	1.70	0.00	1.70	0.00	1.70	0.00	1.70	0.00
	1.00 %	1.70	0.00	1.70	0.00	1.70	0.00	1.70	0.00	1.70	0.00	1.70	0.00
PCA	Control 0%	6.65	0.44	5.80	0.19	5.85	0.63	6.46	0.74	6.15	0.21	4.86	0.34
	0.01 %	6.86	0.26	5.88	0.31	5.80	0.74	6.23	0.53	5.88	0.36	a	a
	0.05 %	5.19	0.56	3.46	0.76	2.40	0.10	4.50	1.96	4.05	0.62	a	a
	0.10 %	4.01	0.64	2.48	0.41	2.00	0.00	2.39	0.48	2.00	0.00	a	a
	0.50 %	2.00	0.00	2.00	0.00	2.00	0.00	2.00	0.00	2.00	0.00	a	a
	1.00 %	b	b	b	b	b	b	b	b	b	b	b	b
GBA	Control 0%	7.65	0.13	7.47	0.13	7.40	0.28	7.55	0.13	7.52	0.13	7.48	0.03
	0.01 %	7.50	0.48	7.35	0.09	7.08	0.13	7.17	0.45	7.15	0.17	7.23	0.13
	0.05 %	6.92	0.40	6.33	0.19	5.80	0.26	6.47	0.14	6.33	0.21	6.42	0.08
	0.10 %	5.63	1.13	5.82	0.74	3.23	1.94	6.05	0.35	4.62	1.21	5.33	0.49
	0.50 %	5.02	0.93	2.68	1.48	2.17	1.16	3.43	1.78	4.55	1.20	1.95	1.03
	1.00 %	3.27	2.63	4.17	0.53	1.97	0.85	1.57	0.98	4.95	1.40	1.88	0.80

SD = Standard deviation CFU = colony forming units SSCA = Stainless steel coupon assay PCA = Plastic coupon assay GBA = Glass bead assay

a = strain not tested b=concentration not included
